# Detection of human pathogenic bacteria in rectal DNA samples from *Zalophus californianus* in the Gulf of California, Mexico

**DOI:** 10.1038/s41598-022-18903-4

**Published:** 2022-09-01

**Authors:** Francesco Cicala, David Ramírez-Delgado, Ricardo Gómez-Reyes, Marcel Martínez-Porchas, Jorge Rojas-Vargas, Liliana Pardo-López, Alexei F. Licea-Navarro

**Affiliations:** 1 Department of Biomedical Innovation, Ensenada Center for Scientific Research and Higher Education, Ensenada, Baja California Mexico; 2 Department of Marine Ecology, Ensenada Center for Scientific Research and Higher Education, Ensenada, Baja California Mexico; 3 Oceanology Research Institute. Autonomous University of Baja California, Ensenada, Baja California Mexico; 4 Laboratory of Experimental Biology, Center for Research in Food and Development, A.C., Hermosillo, Sonora Mexico; 5 Department of Molecular Microbiology, Institute of Biotechnology, National Autonomous University of Mexico, Cuernavaca, Mexico

**Keywords:** Ecological epidemiology, Microbial ecology, Molecular ecology

## Abstract

Human intrusions into undisturbed wildlife areas greatly contribute to the emergence of infectious diseases. To minimize the impacts of novel emerging infectious diseases (EIDs) on human health, a comprehensive understanding of the microbial species that reside within wildlife species is required. The Gulf of California (GoC) is an example of an undisturbed ecosystem. However, in recent decades, anthropogenic activities within the GoC have increased. *Zalophus californianus* has been proposed as the main sentinel species in the GoC; hence, an assessment of sea lion bacterial microbiota may reveal hidden risks for human health. We evaluated the presence of potential human pathogenic bacterial species from the gastrointestinal (GI) tracts of wild sea lions through a metabarcoding approach. To comprehensively evaluate this bacterial consortium, we considered the genetic information of six hypervariable regions of *16S rRNA*. Potential human pathogenic bacteria were identified down to the species level by integrating the RDP and Pplacer classifier outputs. The combined genetic information from all analyzed regions suggests the presence of at least 44 human pathogenic bacterial species, including *Shigella dysenteriae* and *Bacillus anthracis*. Therefore, the risks of EIDs from this area should be not underestimated.

## Introduction

Emerging infectious diseases (EIDs) are defined as diseases that have recently entered into new host populations or increased in incidence or geographic range and include those caused by newly evolved pathogens^1^. Ecological changes act within the background of pathogen evolution in the presence of different strains; however, a key component of the appearance of most EIDs is the ability of microbes to switch between host species^2,3^. Wild animals play direct roles in the evolution of EIDs by acting as pathogen reservoirs and potentiating disease outbreaks^1^. Almost all of the most concerning human pathogens known today have originated in wild animals^4,5^ and are still transmissible from animals to humans (i.e., zoonotic diseases)^6,7^. Notably, in many host species in which zoonotic diseases have originated, the pathogen appears to be harmless with the infected animals showing little or no evidence of clinical disease^8^. However, when these species are transferred from wild animals to humans, the effects of zoonotic diseases can be devastating^7,8^. In fact, zoonotic pathogens are responsible for the most destructive pandemics in human history including the Black Death, Spanish influenza, HIV, and SARS^5,7^.

The rise in zoonotic diseases is thought to be driven by complex interactions between diverse socio-economic, environmental, and ecological factors^2,3^. Human intrusions into undisturbed wildlife is considered to be among the major contributors to the evolution of novel zoonotic diseases^4,9^. In this context, the Gulf of California (GoC) is an example of an undisturbed ecosystem that has only recently experienced human intrusion. At present, human population density along the western coast of the GoC is relatively low but is rapidly increasing along the eastern coast^10^. The rapid intensification of human activities and the accompanying environmental changes may increase the occurrence of novel EIDs. Thus, the presence of potential human pathogen in this ecosystem requires appropriate researches.

Sentinel species that are also top marine predators have been previously proposed as suitable tools to estimate environmental change^11^. These species may integrate the information from an ecosystem at multiple trophic levels and thus may be helpful in identifying potential risks to human health^11,12^_._ The California sea lion (*Zalophus californianus*) in the GoC has been proposed as the main sentinel species in the region. These pinnipeds are top predators in the gulf, live in geographically distinct colonies, and exhibit similar food preferences to humans^13^. The gastrointestinal (GI) microbial communities in California sea lions are likely to include human pathogenic bacteria. For example, previous studies have shown that sea lion spp. bacterial communities may trigger numerous zoonotic diseases including gastritis (*Helicobacter* spp.), bacterial dermatitis, and conjunctivitis^13–15^. Due to frequent close encounters with humans given the booming and largely unsupervised pinniped tourism industry, sea lions may act as links between wild animals and humans^16^. For instance, the increase in human-sea lion interactions has led to an increase in the number of human zoonotic diseases that are mostly induced by bacterial species such as *Vibrio parahemolyticus* and *Staphylococcus aureus*^13,17^. Accordingly, assessing the GI microbiomes of California sea lions in the GoC may provide insights into the risks of EIDs and re-emerging zoonotic diseases.

Recent advances in sequencing technology have produced new and powerful tools to assess microbial biodiversity from almost any source of environmental DNA, including samples collected from the GI tracts of host species. Metabarcoding, the taxonomic characterization of environmental communities through the short DNA sequences of one gene, is considered one of the most powerful tools to comprehensively evaluate bacterial communities^18^. Metabarcoding studies usually involve sequencing a fragment of the *16S rRNA* gene, which is composed of nine conserved and hypervariable regions (V1 to V9)^19^. Conventionally, conserved regions are used to design universal primers while the genetic information of hypervariable regions facilitates the characterization of bacterial consortia^19^. However, metabarcoding has several limitations that may lead to data being misinterpreted. For example, there is currently no consensus regarding which of the nine *16S rRNA* hypervariable regions should be targeted to assess bacterial communities^20,21^. Consequently, the analysis of different hypervariable regions may limit the inferences that can be made between studies^20^. In addition, different sets of primers may exhibit variable affinities for different bacterial taxa that result in unequal PCR amplification and a limited capacity to detect ecologically important bacteria groups^22^. These limitations related to the hypervariable regions prompted Fuks and colleagues to propose the Short Multiple Regions Framework (or SMURF), which compares information from different *16S rRNA* regions to overcome the aforementioned limitations^19^.

In the current study, metabarcoding surveys were conducted to identify human pathogenic bacterial species from the GI tracts of wild sea lions and evaluate the risk of potential zoonotic EIDs in the GoC. Overall, a total of 36 DNA-pool samples were analyzed from rectal specimens collected from sea lion pups distributed in geographically distinct rookeries across the GoC. To conduct the most accurate characterization of GI bacterial communities possible, a SMURF approach was adopted using the Ion 16S Metagenomic Kit™ (Catalog no. A26216 Life Technologies, Grand Island, NY) designed for the Ion Torrent Personal Genome Machine (Life Technologies, Grand Island, NY). The kit includes six proprietary primer sets that target seven hypervariable regions of *16S rRNA*. As the outputs from V6 and V7 are combined, these regions are denoted in this study as V6-7. In addition, an in-house analytical pipeline is proposed to incorporate open access bioinformatics platforms and programs in downstream analyses.

## Results

### Data summary

In total, 36 rectal DNA-pool samples were sequenced and 304,536 *16S rRNA* reads were returned with average lengths of 241 bp. After performing quality control tests and excluding chimeras or rare ASVs, 203,754 reads were retained. As we previously reported, the number of reads and detected ASVs varied according to which hypervariable region was analyzed^23^. Specifically, the number of detected ASVs and reads (included in parentheses) were 189 (22,139), 207 (40,167), 177 (19,428), 210 (70,059), 161 (22,984), and 14 (28,977) for V2, V3, V4, V67, V8, and V9, respectively. Despite the variations between the number of detected ASVs and read numbers, the asymptotic shapes of the rarefaction curves suggest that the major fraction of bacterial diversity was detected using V2, V3, V4, V67, and V8 regions. However, the ASVs detected for V9 were only assigned to *Protobacteria* (Fig. S1).

### Taxonomic classification and human pathogenic bacteria

Complementary taxonomies were obtained by RDP and Pplacer. Early taxonomic positions were assigned to ASVs by RDP; however, Pplacer was used to further identify and medically sort important bacterial species. Specifically, RDP classified the majority of ASVs to higher taxonomic levels (e.g., order and family). Lower taxonomic level classifications of ASVs were challenging for RDP, and averages of 65.6% and 2.95% of reads were assigned to genus and species levels, respectively. Conversely, Pplacer classified 72 ASVs, which represented 55.7% of the total number of reads (from Tables S2 to S7), to the species level. As expected, the Pplacer classification was only successful for known bacterial species. Phylogenetic reconstructions revealed that several ASVs exhibited monophyletic relationships with pathogenic genera, such as *Actinobacillus*, *Campylobacter*, *Capnocytophaga*, *Klebsiella*, and *Streptococcus*, which suggests that the GI tracts of sea lions may still contain unknown and medically important bacterial species (from Figs. S2 to S7).

**Figure 1:**
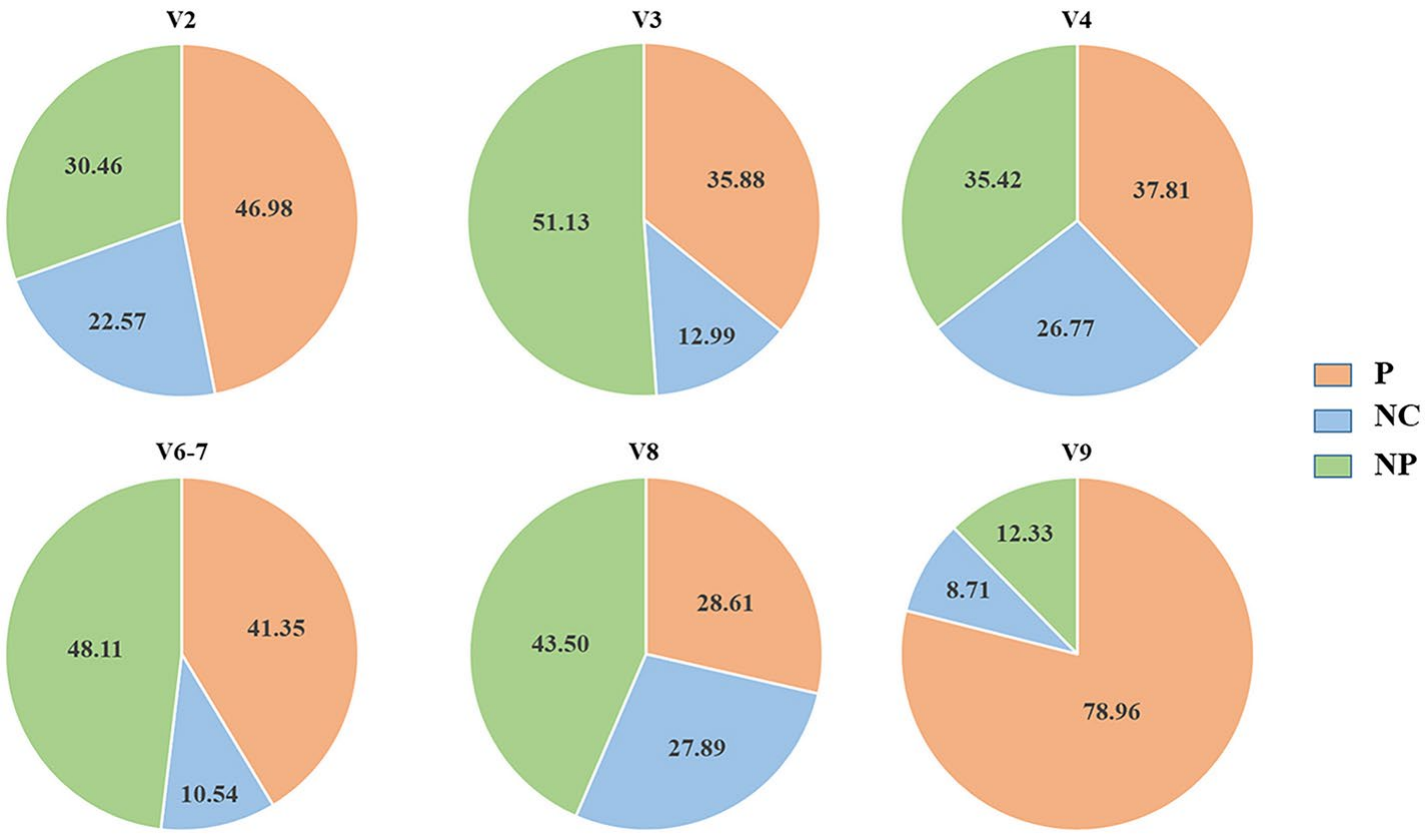
Proportions of “*potential pathogenic bacteria*” (P) which was defined with ASVs with the same (e.g., at the genus level in both RDP and Pplacer) or complementary (e.g., at family level in RDP and genus and species levels in Pplacer) taxonomic assignments from both classifiers; (2) “*no consistent pathogenic bacteria*” (NC) which was defined as ASVs with discordant taxonomic classifications between classifiers; and (3) “*no pathogenic bacteria*” (NP) detected among the analyzed hypervariable regions (V2 to V9).

Data from both RDP and Pplacer revealed 44 human pathogenic bacterial species assigned to 26 genera (Table 1). In relation to the number of reads, these findings suggest that 45% of the entire GI bacterial microbiota of sea lions is potentially pathogenic to humans. Subsequently, 37% and 18% of the reads were assigned to the “*no pathogenic bacteria*” and “*no consistent pathogenic bacteria*” groups, respectively. Once again, the exact amount of ASVs varied among 16S hypervariable regions (Fig. 1; from Tables S2 to S7).

**Table 1:** List of detected human pathogenic bacterial species, hypervariable regions of detection, risk level groups (according to the TRBA 466prokaryotic list), and GenBank access numbers.

Species	Region	TRBA 466 risk group	TRBA 466 tags	GenBank access number
*Actinobacillus equuli*	V8	2	ht	NR_118760.1
*Actinobacillus suis*	V8	2	ht	M75071.1
*Alistipes putredinis*	V2, V3, V4, V6-7	2	ht	LT223618.1
*Anaerobiospirillum thomasii*	V2, V3, V4, V8	2	ht	NR_025518
*Anaerobiospirillum succiniciproducens*	V8	2	ht	NR_026075
*Arcanobacterium haemolyticum*	V2, V3, V4, V8	2	ht	NR_074602.1
*Arcanobacterium pyogenes*	V6–7	2	ht	EU268192.1
*Bacillus anthracis*	V3, V6–7	3	Z	AY138383.1
*Bacteroides fragilis*	V6–7	2	TA, ht	KP326374.1
*Bacteroides tectus*	V2, V3, V4	2	ht	GQ422748.1
*Bergeyella zoohelcum*	V3	2	Z	NR_104718.1
*Campylobacter fetus*	V3	2	Z	AF482990.1
*Campylobacter jejuni*	V3	2	Z	Y19244.1
*Campylobacter lari*	V2	2	Z	NR_042683.1
*Campylobacter rectus*	V2, V4, V8	2	Z	NR_113247.1
*Campylobacter upsaliensis*	V2, V6–7, V8	2	Z	AB980278.1
*Campylobacter ureolyticus*	V3	2	ht	FN401327.1
*Capnocytophaga cynodegmi*	V6–7	2	ht	KT194087.1
*Clostridium fallax*	V3	2	ht	NR_044714.2
*Enterobacter cloacae*	V4	2	ht	JF894166.1
*Escherichia albertii*	V2, V4, V6–7	2	ht	MT982723.1
*Escherichia coli*	V2, V4	2	TA, Z, ht	JF895181.1
*Escherichia fergusonii*	V3, V8	2	ht	MF973086.1
*Fusobacterium necrophorum*	V6–7, V8	2	ht	AM905356.1
*Fusobacterium varium*	V2, V4	2	ht	NR_113384.1
*Helicobacter cinaedi*	V2, V4	2	ht	NR_025941.1
*Klebsiella oxytoca*	V6-7	2	ht	KF145193.1
*Moraxella osloensis*	V2, V4	2	ht	NR_104936.1
*Mycoplasma felis*	V6–7, V8	2	Z	NR_029174.1
*Neisseria meningitidis*	V8	2	V	DQ201319.1
*Parvimonas micra*	V3, V4	2	ht	NR_114338.1
*Porphyromonas asaccharolytica*	V6–7	2	ht	NR_074588.1
*Porphyromonas gingivalis*	V2, V4	2	ht	AB910743.1
*Porphyromonas levii*	V3, V6–7	2	ht	AB547664.1
*Pseudomonas aeruginosa*	V8	2	ht	JN995662.1
*Psychrobacter osloensis*	V3	2	ht	NR_104936.1
*Salmonella enterica*	V9	2	Z	NR_044370.1
*Shigella dysenteriae*	V2, V4, V6–7, V8	3	T	EU009182.1
*Streptococcus dysgalactiae*	V4	2	ht	EU660339.1
*Streptococcus equi*	V8	2	ht	MF155598.1
*Streptococcus equinus*	V3	2	ht	MF480438.1
*Streptococcus gallolyticus*	V2, V4, V6–7, V8	2	Z	AF323911.1
*Streptococcus intermedius*	V6–7	2	ht	GU045403.1
*Streptococcus porcinus*	V8	2	ht	DQ303195.1

*ht* pathogen for humans and vertebrates, *Z* zoonotic pathogen, *T* toxin production, *TA* species for which strains are known and have been handled safely, *V* effective vaccine available.

### Bacterial diversity among rookeries and hypervariable regions

**Figure 2:**
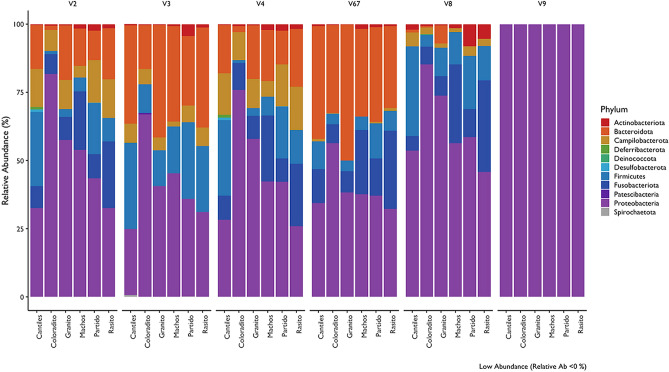
Major bacterial taxa (> 1% in terms of read abundance) of the gastrointestinal (GI) microbiota samples collected from six breeding rookeries (Rasito, Partido, Machos, Granito, Coloradito, and Cantiles) in the Gulf of California (GoC). The DNA samples were pooled (see methods) and taxa were detected from each hypervariable region (V2 to V9) of *16S rRNA.*

**Figure 3:**
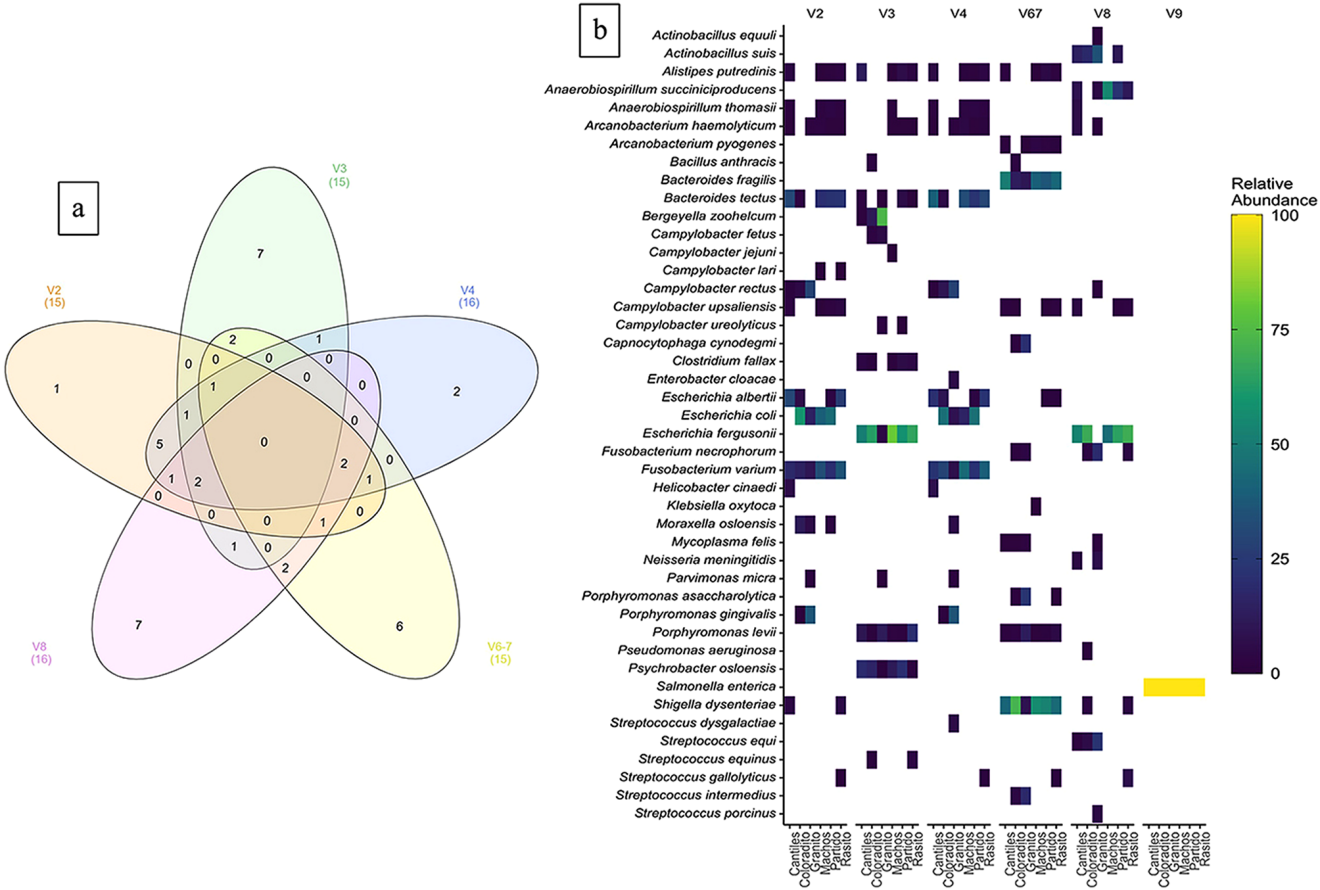
Number of exclusive or shared human pathogenic bacteria among the analyzed hypervariable regions (V2 to V9) (**a**) and geographic areas (i.e., rookeries) (**b**). To facilitate Venn diagram interpretation, the V9 region was not included. However, an additional exclusive human pathogenic species, *Salmonella enterica*, should only be considered for the V9 region. The heat map is based on a log2 transformation of the relative abundances of the pathogenic species detected for each hypervariable region (V2 to V9) and between all sampled rookeries (Rasito, Partido, Machos, Granito, Coloradito, and Cantiles).

Bacterial diversity was evaluated to identify patterns in GI microbiome composition according to the hypervariable regions and geographic area (e.g., among rookeries). Based on the average read frequency (included in parentheses) at the phylum level, the RDP classification revealed *Protobacteria* (up to 85%) to be the predominant bacterial group among all hypervariable regions, followed by *Bacteroidota* (up to 32%) and *Firmicutes* (up to 15%). Distinctive arrangements were also observed among hypervariable regions. For example, *Desulfobacterota* was mainly detected with V3, while *Campilobacterota* was mainly detected with V2 and V4 (Fig. 2). Similar patterns were also observed for medically important species. Indeed, the combined data from both the RDP and Pplacer classifications revealed that most human pathogenic bacteria that were detected were exclusive to a 16S hypervariable region (Fig. 3a,b). For example, *Salmonella enterica* was only detected with V9 while the majority of *Campylobacter* species were detected with either V3 or V8 (Table 1). The maximum number of shared ASVs (n = 5) was observed between the V2 and V4 regions (Fig. 3a). Also, based on the average number of implemented ecological indexes, the highest bacterial community diversity was detected with V3 (number of ASVs: 64.33; Shannon: 5.05), while the least complex bacterial community was detected with V9 (number of ASVs: 7.12; Shannon: 1.5).

Obvious geographical patterns were not observed (e.g., the presence or absence of a given bacterial species among rookeries) according to the abundance of bacterial reads detected among rookeries (Fig. 3b); however, bacterial diversity appeared to be greater in the middle-southern rookeries, as higher numbers of ASVs were observed among Cantiles and Partido (Fig. S1).

## Discussion

Sequence analysis of housekeeping genes, such as the bacterial *16S rRNA* gene, is increasingly being used to identify medically important bacterial species in both clinical practice and scientific investigations^24^. Recent advances in high throughput sequencing have provided unprecedented opportunities to assess the entire microbial community from almost any environmental DNA sample, including the GI tracts of host species. However, accurate descriptions of these communities can be difficult to obtain due to the unequal amplification of bacterial taxa during PCR^22^ and the inability to meet the genus-species taxonomic binomial for the majority of sequences^25^. This study attempted to overcome these limitations by employing a *Short Multiple Regions Framework* (SMURF) approach using the 16S Metagenomics sequencing kit for Ion Torrent that allows for the simultaneous amplification of six hypervariable regions of the *16S rRNA* gene. Next, RDP (a naive Bayesian taxonomic classifier method;^26^) and Pplacer (a likelihood-based phylogenetic inference;^25^) classifiers were adopted to identify human pathogenic bacteria down to the species level.

The findings from this study support SMURF as the preferred approach in metabarcoding surveys to characterize bacterial communities. Indeed, distinctive region-related bacterial arrangements were observed with each analyzed *16S rRNA* fragment. These findings are consistent with previous publications where a single-locus approach has been found to restrict detection of certain bacterial species, alter relative abundances, and lead to misleading interpretations of microbiome composition^22,27^. Based on our experience with bioinformatics classification tools, we considered that RDP and Pplacer could provide complementary outcomes. We deemed RDP to be appropriate, as this tool does not require a priori taxonomic knowledge of reads given that its taxonomic identification relies on unambiguous matching (or exact genetic similarity) between query and reference sequences with assigned nomenclature^26^. For instance, during RDP classification, all query reads are trained on all reference sequences that are present in a chosen database. The nomenclature of the most similar reference sequence is then used to infer the taxonomic information of queries. However, although RDP is a rapid and accurate method for taxonomic classification in many environments, only a few sequences will be assigned to the species level, as reference databases are known to be incomplete, which limits the ability to classify reads at low taxonomic levels^28^. Further limitations may arise with RDP when a chosen marker does not have the genetic resolution for species-level identification. In these cases, multiple reference sequences will share the same DNA-sequence, and it will be impossible to accurately assign the taxonomic position of a read^28^.

Conversely, Pplacer is a software package for the likelihood-based phylogenetic placement of a read on a reference tree. Likelihood-based phylogenetic inferences are generally considered to be the most reliable classification methods for unknown sequences^25^, even though outcomes strongly rely on the a priori assumptions that are needed to build adequate reference trees. This method usually allows for only a few reads to be identified that are related to the known species used in phylogenetic reconstructions. The identification of such low numbers of reads may not be suitable for most metarbarcoding surveys that consist of thousands of unidentified taxonomic reads. As has been previously reported for RDP, closely related reference sequences or a lack of genetic resolution may lead to inaccurate species-level identification. In this study, high genetic similarity was observed among Pplacer reference sequences that may have contributed to a false-positive detection of pathogenic species (as inconsistent results were observed with RDP). Thus, to prevent overestimating human pathogenic bacteria, or at least to minimize such bias, the RDP and Pplacer outcomes were compared.

To the best of our knowledge, no studies have examined GI bacterial communities in rectal DNA samples collected from sea lions through a metabarcoding approach with six hypervariable regions and two independent bioinformatic classification tools. Therefore, the findings from this novel study may assist in evaluating the hazards associated with EIDs in the GoC. By first characterizing the GI tracts of sea lions throughout the GoC, our findings suggest that this pinniped (and probably other host organisms in the GoC) may harbor at least 44 potential human pathogenic bacterial species. Notably, the absence of geographical patterns between sampling areas suggests that the identified bacterial species are uniformly distributed along the GoC. Among the medically important species identified in this study, *Shigella dysenteriae* and *Bacillus anthracis* appear to be the most concerning bacteria, as these two species are classified in Risk Group 3 of the TRBA-466 prokaryotes classificationhttps://paperpile.com/c/zF6cMX/YbbY. For practical purposes, the biological agents in this list are grouped into four levels (1 to 4) that reflect low (group 1) and high (group 4) probabilities of these agents causing infectious disease in humans.

*Shigella* was detected as a bacterial agent in this study that was grouped into ABAS level 3. This genus is composed of Gram-negative and facultative anaerobic bacteria that are currently included in the *Enterobacteriaceae* family. The exact taxonomic composition of this family is up for debate, and recent molecular phylogenetics studies have suggested that *Enterobacteriaceae* should be divided into multiple families^29–31^. Our findings support the observation that the global range of *Enterobacteriaceae* is much more extensive than previously thought^31^. Members of this family play important roles in vegetative processes in the environment; however, some studies have recently proposed that some previously established species may trigger infectious diseases and syndromes in humans^29,31^. At present, the *Shigella* genus includes *S. dysenteriae*, *S. flexneri*, *S. boydii*, and *S. sonnei*^32^. This genus has evolved highly invasive systems to invade and multiply within the intestinal epithelia of humans and other hosts to cause severe inflammatory colitis, which is commonly called Shigellosis^33^. Shigellosis remains a worldwide health concern because it is responsible for diarrhea-associated morbidity and mortality across all ages and is the second leading cause of death in children under the age of 5 years^34,35^. Conventionally, the natural hosts of *Shigella* are humans and other primates; however, reports of *Shigella* infection in new hosts, including monkeys, rabbits, calves, piglets, and even chickens, have been recently published^32,36,37^.

*Bacillus anthracis* is a spore-forming, Gram-positive bacterium responsible for anthrax, an acute zoonotic disease of almost all mammals. Anthrax can be fatal to livestock, wild animals, and humans^38–40^. There are numerous unknown factors that influence the epidemiology of anthrax in multi-host systems, especially at wildlife-human interfaces^38^. Despite decades of research, the geographical distribution of *B. anthracis* is still poorly understood, which has resulted in many countries having limited or inadequate surveillance systems^39^. Finally, the detection of *S. dysenteriae* and *B. anthracis* in sea lions suggests that these pathogenic bacterial species may inhabit a greater number of host species than previously thought. This observation suggests that pinnipeds should be considered potential carriers and disseminators of certain bacterial genera or species.

In addition to these well-established pathogens, we detected several bacterial species of growing concern that are currently classified as “high priority pathogens” by the World Health Organization. In this context, *Campylobacter jejuni* is considered to be the main pathogen involved in human food-borne outbreaks worldwide^41^. *Campylobacter jejuni* is a Gram-negative, aerobic, pleomorphic, and mobile bacterium. The taxonomy of the *Campylobacter* genus has changed dramatically since its discovery^41,42^. At present, this genus comprises 25 species, 2 candidates species, and 8 subspecies, many of which are of clinical and economic importance^41^. Among them, *C. jejuni* is one of the most medically important species and has been recognized since 1970 as the predominant bacterium of gastroenteritis, although it is associated with miscarriages and involved in other diseases such as proctitis, septicaemia, and meningitis^41,43^. Notably, in several livestock species, such as chickens, dogs, pigs, and sheep, *C. jejuni* is considered to be commensal organism^44^. Our findings suggest that *C. jejuni* may also be a commensal bacteria in wild sea lions. *Bacteroides fragilis* has been previously reported as the most frequent anaerobic pathogen isolated from human GI infections^45^. At the moment, the *Bacteroides* genus includes 22 species, most of which have been isolated from human feces^46^. *Bacteroides fragilis* is the most common anaerobe and pleomorphic bacterium responsible for endogenous infections, particularly those of the abdominal cavity, with associated mortalities of more than 19%^45–47^. *Bacteroides fragilis* (as well as other *Bacteroides* species) may escape the intestine after the integrity of the intestinal wall becomes distorted to infect soft tissues in which they may promote several inflammatory conditions or diseases such as brain abscesses or toxin-associated diarrhea^45,48^.

Based on these examples, it is possible that host shifts have frequently occurred during the evolution of these bacteria; however, further research is needed to validate or reject this hypothesis. It is also apparent from the phylogenetic reconstructions of this study that yet undiscovered and medically important species may be harbored by the California sea lions of the GoC. Again, more research is needed to determine the exact taxonomic positions and risks associated with these potential human pathogenic bacterial species.

## Methods

### Sample collection

**Figure 4:**
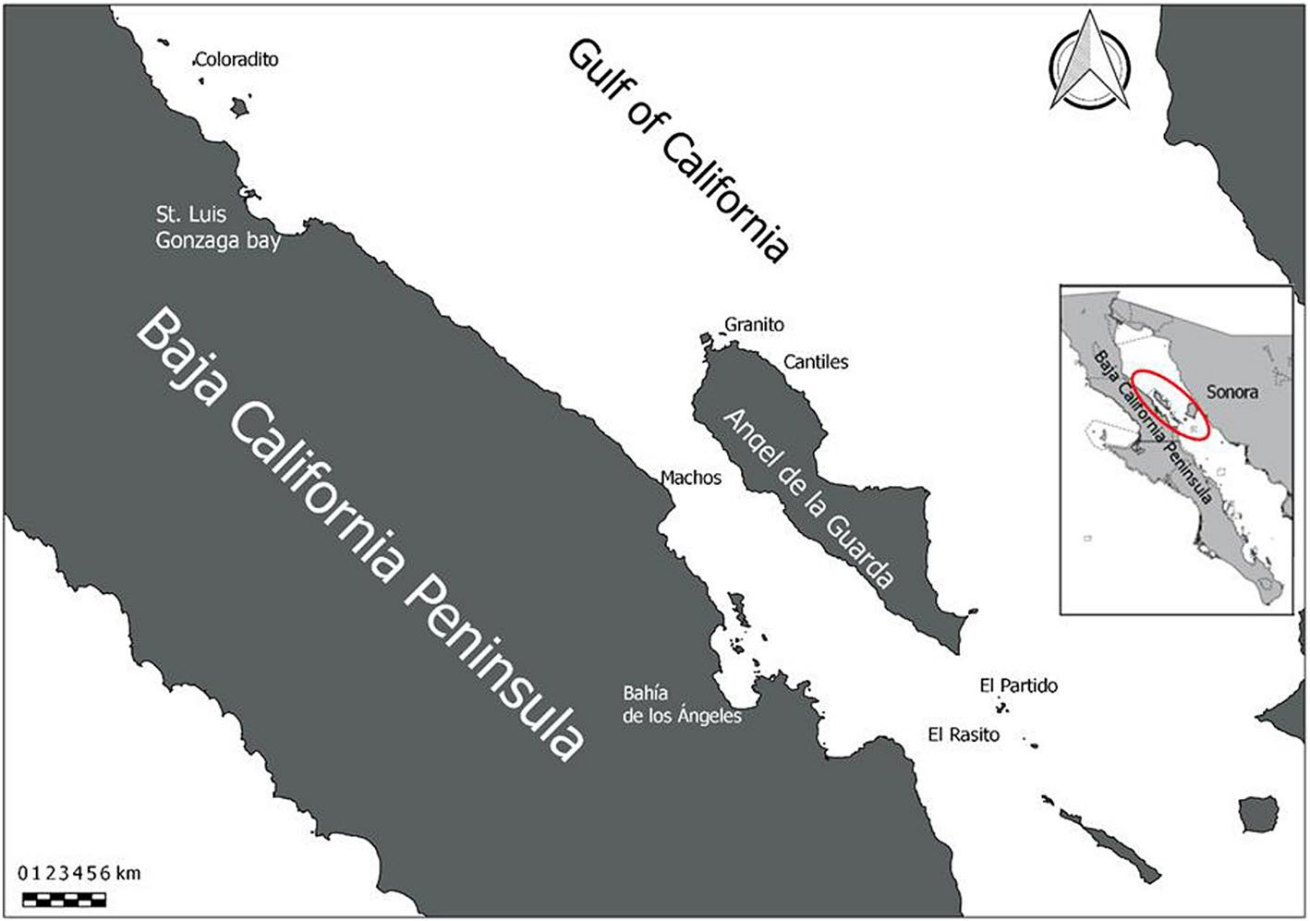
Islands Region in which the six sampled sea lion rookeries were located in the Gulf of California (GoC). The rookies included those of Coloradito, Granito, Cantiles, Machos, Partido, and Rasito.

A total of 70 rectal cotton swabs (RCSs) were collected over two field expeditions that occurred from June–August of 2018 and 2019. Samples were collected from sea lion pups (2–3 months old) in six rookeries along the GoC [from Coloradito (30° 02′ N–114° 29′ W) to Rasito (28° 50′ 12″ N–112° 59′ 56 W); Fig. 4]. These samples were collected in compliance with the regulations of the Mexican government (SGPA/DGVS/003086/18). Wild sea lion pups were manually restrained and anesthetized with 5% isoflurane to reduce animal mobility and alleviate sensations of pain. Anesthetic agents were administered with the assistance and approval of African Safari Zoo (Puebla, Mexico). After collection, the RCSs were immediately preserved in liquid nitrogen until DNA extraction.

### DNA extraction and Ion Torrent sequencing

Total DNA was extracted and purified from the preserved FCSs using both the Wizard Genomic DNA purification kit (Promega Corporation, Madison, WI, USA) and the PureLink Invitrogen Genomic DNA kit (Thermo Fisher, Carlsbad, CA, USA) following manufacturer protocols. DNA pools were prepared by combining the extracted DNA (30 nM) of three randomly selected RCSs from the same rookery and year. DNA extracts were pooled to increase test sensitivity. A total of 36 DNA pools were prepared before being amplified with the Ion 16S Metagenomics kit (Thermo Fisher Scientific, Waltham, USA) following manufacturer protocols. Two primer sets were employed for PCR; the first set contained primers targeting the V2, V4, and V8 regions, while the second set targeted the V3, V6–7, and V9 regions. Amplification involved 25 PCR cycles and Ion Xpress Barcoded adapters. Emulsion PCR used OneTouch™2 400 bp read length chemistry, and sequencing was performed on an Ion Torrent PGM with Torrent Suite v. 4.2.1.

### Sequence curation, quality control analyses, and library preparation

Ion Reporter Metagenomics 16S v. 5.2 was used to initially trim Ion adapters, barcode, primers and to filter reads according to their quality scores (Q). Reads with Q < 20 were discarded prior to further analysis. Raw DNA libraries from the same rookery were then joined and deposited at NCBI under the NCBI BioProject number PRJNA761728.

DNA libraries were then trimmed in *single-end-mode* using trimmomatic v. 0.38^49^ with the following parameters: AvgQual 25, Leading 3; Trailing 3; Slidingwindow 4:15, and Minlen 150. In addition, reads that met this early quality control test were filtered and denoised using the plugin DADA2 v. 1.16^50^ in RStudio v. 1.1.463 (R Core Team, 2016). DADA2 v. 1.16 was also used to detect and remove chimeras. Finally, to minimize the chance of including spurious amplicon sequence variants (or ASVs) in the final datasets, ASVs with < 15 total reads were excluded before generating both the representative sequences and feature table files. Next, the ASVs were separated into their respective regions of *16S rRNA* because the 16S Metagenomics Kit simultaneously amplifies six different variable regions for each sample. To achieve this, representative sequences were aligned against the *16S rRNA* sequences of *Streptococcus mutans* (Genbank Accession: DQ677761)^20^ using MAFFT v. 7.313^51^ by implementing the*—addfragments* option, which successively adds unaligned sequences or short DNA fragments into an existing multiple sequence alignment^52^. Boundaries between variable regions were visualized in MEGA v. X^53^, and the ASV libraries from each hypervariable region were exported separately.

To evaluate how exhaustively the bacterial communities of each hypervariable regions were sampled, rarefaction curves of the detected ASVs were generated using the QIIME 2^54^ *diversity alpha-rarefaction* plugin, and the median frequency of the reads was set as the sequencing depth. Both alpha- and beta-diversity were determined using the rarefied number of reads as a proxy for abundance in QIIME 2.

### Taxonomic assignments and the identification of potential pathogenic bacterial species

Based on our knowledge and previous experience overcoming the methodological limitations of the most common informatics classifier, potential human pathogenic bacteria were identified up to the species level using two complementary bioinformatics software. Initially, a taxonomic classification of all detected ASVs was conducted via the RDP classifier in Dada2 v. 1.16 that was trained against the SILVA v. 138 database^55^ implementing the *addSpecies* function. This function was enabled to increase taxonomic confidence^56^ and extend the assignment of representative sequences beyond the genus level^50^. The ASVs with unassigned families (or higher taxonomic levels) were not further investigated as possible pathogenic bacteria and were not considered to be pathogenic (as described below).

The identification of potential human pathogenic bacterial species was achieved with the Pplacer v. 1.1^25^ program suite. Briefly, these programs place query sequences into reference phylogenetic trees to classify species and linages^25^. RDP classification was then used to sort only pathogenic bacterial genera according to the TRBA-466 prokaryote classification [Ausschuss für Biologische Arbeitsstoffe (ABAS), 2015/2015]^57^. High quality *16S rRNA* sequences (1480–1530 bp) of human pathogenic species of selected genera were downloaded from NCBI GenBank^58^ and employed as reference sequences in successive phylogenetic analyses. Query and reference sequences were aligned using MAFFT v. 7.313^51^ and placed in a reference tree built with the Maximum Likelihood method using RAxML v. 8.2.10^59^ under the General Time Reversible (GTR) model and discrete gamma distribution (GAMMA)^59^. Notably, distinct bacterial microbiota arrangements were observed among hypervariable regions, so specific reference sequences were prepared ad hoc for each *16S rRNA* region analyzed and are reported in Table S1.

Finally, taxonomic assignments for the ASVs obtained via RDP and Pplacer were compared, and three discrete categories were identified: (1) “*potential pathogenic bacteria*,” which was defined with ASVs with the same (e.g., at the genus level in both RDP and Pplacer) or complementary (e.g., at family level in RDP and genus and species levels in Pplacer) taxonomic assignments from both classifiers; (2) “*no consistent pathogenic bacteria*,” which was defined as ASVs with discordant taxonomic classifications between classifiers; and (3) “*no pathogenic bacteria*,” which was defined as no human pathogenic species or ASVs with species-level taxonomies that were not defined with either the RDP or Pplacer approaches. Shared or medically exclusive and important ASVs among hypervariable regions were visualized using Venn diagrams created with the open web-software InteractiVenn^60^.

### Ethics declarations

Gastrointestinal samples were collected following the regulations of the Mexican government, Bioethical committee of the agriculture and wild life agency (SGPVA/DGVS/003083/18) and with the assistance and approval of African Safari Zoo (Puebla, México). The study is reported in accordance with ARRIVE guidelines.

## Supplementary Information

Supplementary Figures.

Supplementary Tables.

## Data Availability

Raw reads were deposited in the National Center for Biotechnology Information (NCBI) under the BioProject PRJNA761728 (https://www.ncbi.nlm.nih.gov/bioproject/PRJNA761728/)).
