# Supplementary material for: Detection of human pathogenic bacteria in rectal DNA samples from Zalophus californianus in the Gulf of California, Mexico
Source: Sci Rep. 2022 Sep 1;12:14859. doi: 10.1038/s41598-022-18903-4 (PMC9434536; doi:10.1038/s41598-022-18903-4)
Supplement: Supplementary file 1 — Supplementary Figures. [file 41598_2022_18903_MOESM1_ESM.pdf]

Article title

Detection of human pathogenic bacteria in fecal DNA samples from *Zalophus californianus* in the Gulf of California, Mexico

Author names and affiliations

Francesco Cicala [1], David Ramírez-Delgado [2], Ricardo Gómez-Reyes [3], Marcel Martínez-Porchas [4], Jorge Rojas-Vargas [5], Liliana Pardo-López [5] and Alexei F. Licea-Navarro [1*].

1. Department of Biomedical Innovation, Ensenada Center for Scientific Research and Higher Education, Ensenada, Baja California, Mexico.
2. Department of Marine Ecology, Ensenada Center for Scientific Research and Higher Education, Ensenada, Baja California, Mexico.
3. Oceanology Research Institute. Autonomous University of Baja California, Ensenada, Baja California, Mexico.
4. Laboratory of Experimental Biology, Center for Research in Food and Development, A.C., Hermosillo, Sonora, Mexico.
5. Department of Molecular Microbiology, Institute of Biotechnology, National Autonomous University of Mexico, Cuernavaca, Mexico.

* Correspondence: alicea@cicese.mx

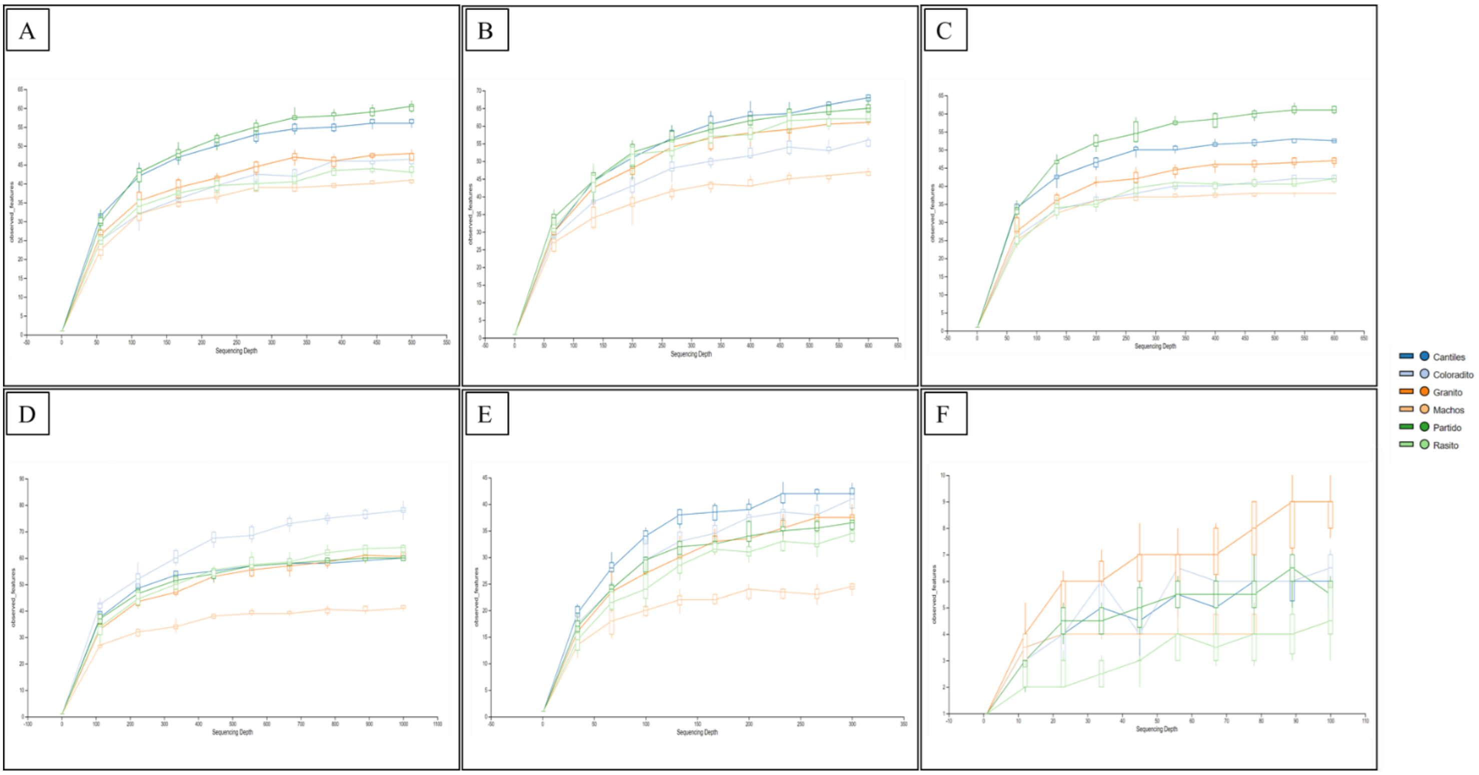

Figure S1: Rarefaction curves based on the cumulative number of observed amplicon sequence variants (ASVs) for V2, V3, V4, V6-7, V8, and V9 hypervariable regions reported as A, B, C, D, E, and F, respectively.

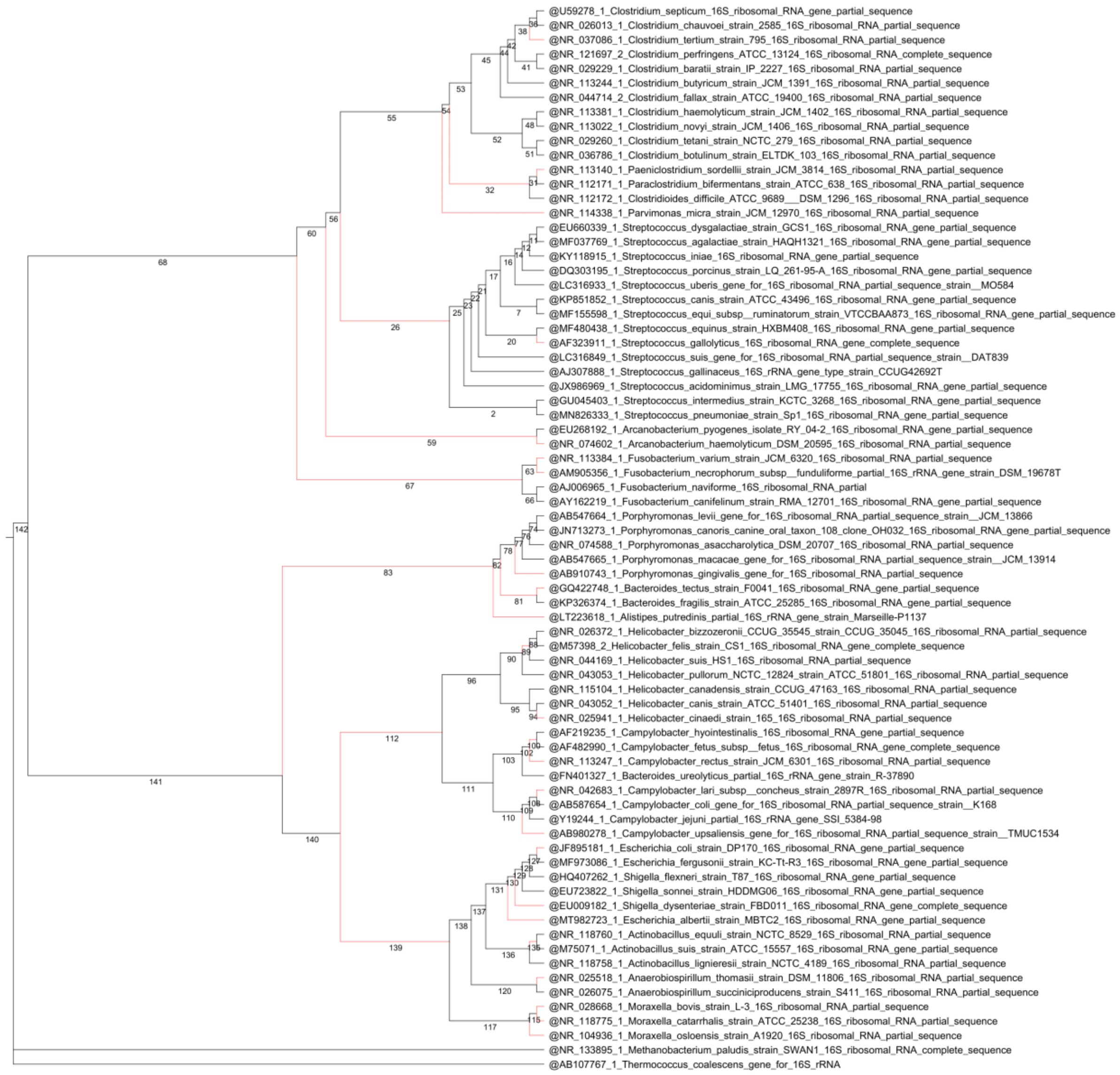

Figure S2: Phylogenetic insertion tree produced by PPLACER using the posterior probability method showing the phylogenetic placements of the sequences obtained with the V2 hypervariable region of the *16S rRNA* gene.

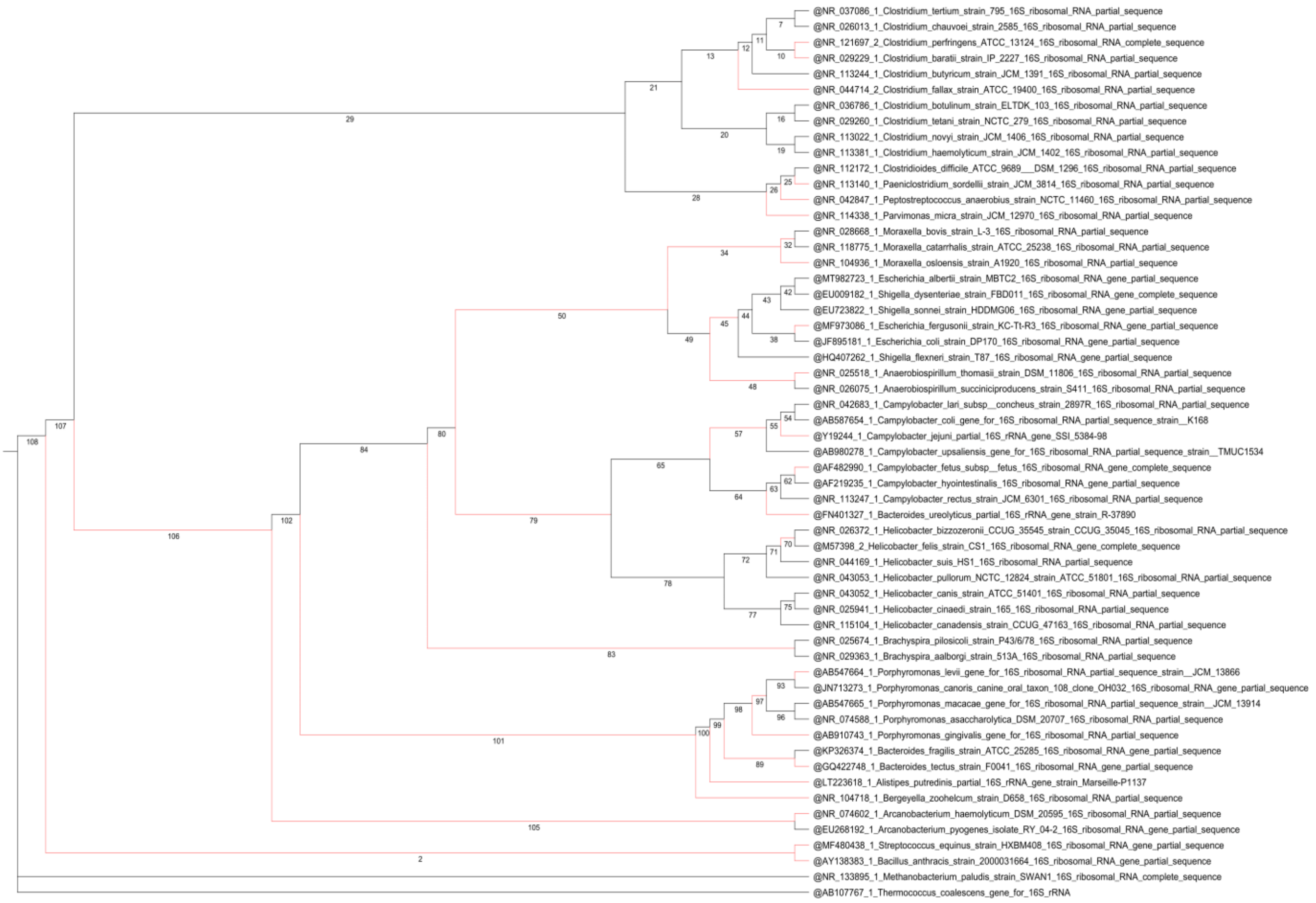

Figure S3: Phylogenetic insertion tree produced by PPLACER using the posterior probability method showing the phylogenetic placements of the sequences obtained with the V3 hypervariable region of the *16S rRNA* gene.

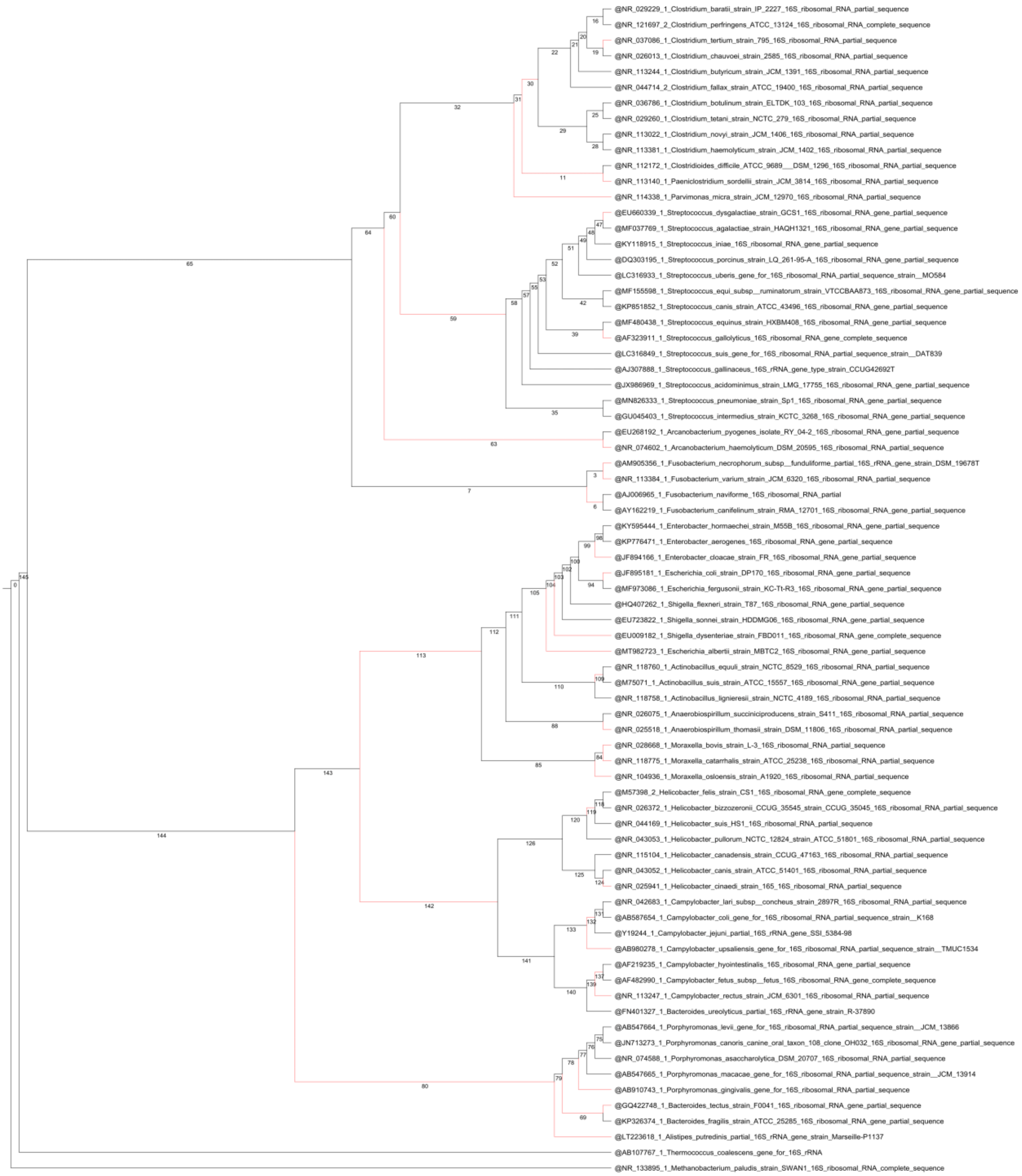

Figure S4: Phylogenetic insertion tree produced by PPLACER using the posterior probability method showing the phylogenetic placements of the sequences obtained with the V4 hypervariable region of the *16S rRNA* gene.

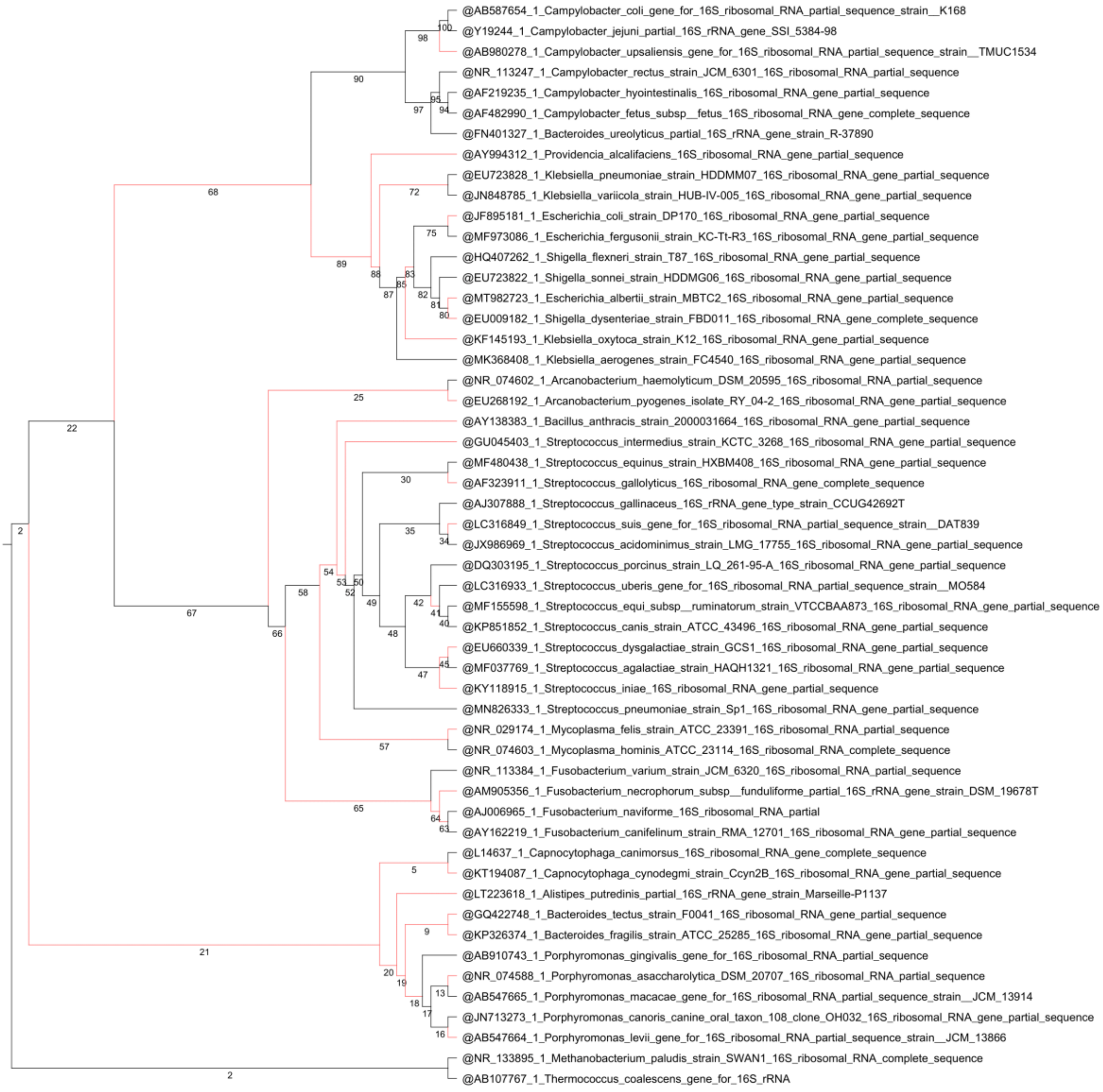

Figure S5: Phylogenetic insertion tree produced by PPLACER using the posterior probability method showing the phylogenetic placements of the sequences obtained with the V6-7 hypervariable region of the *16S rRNA* gene.

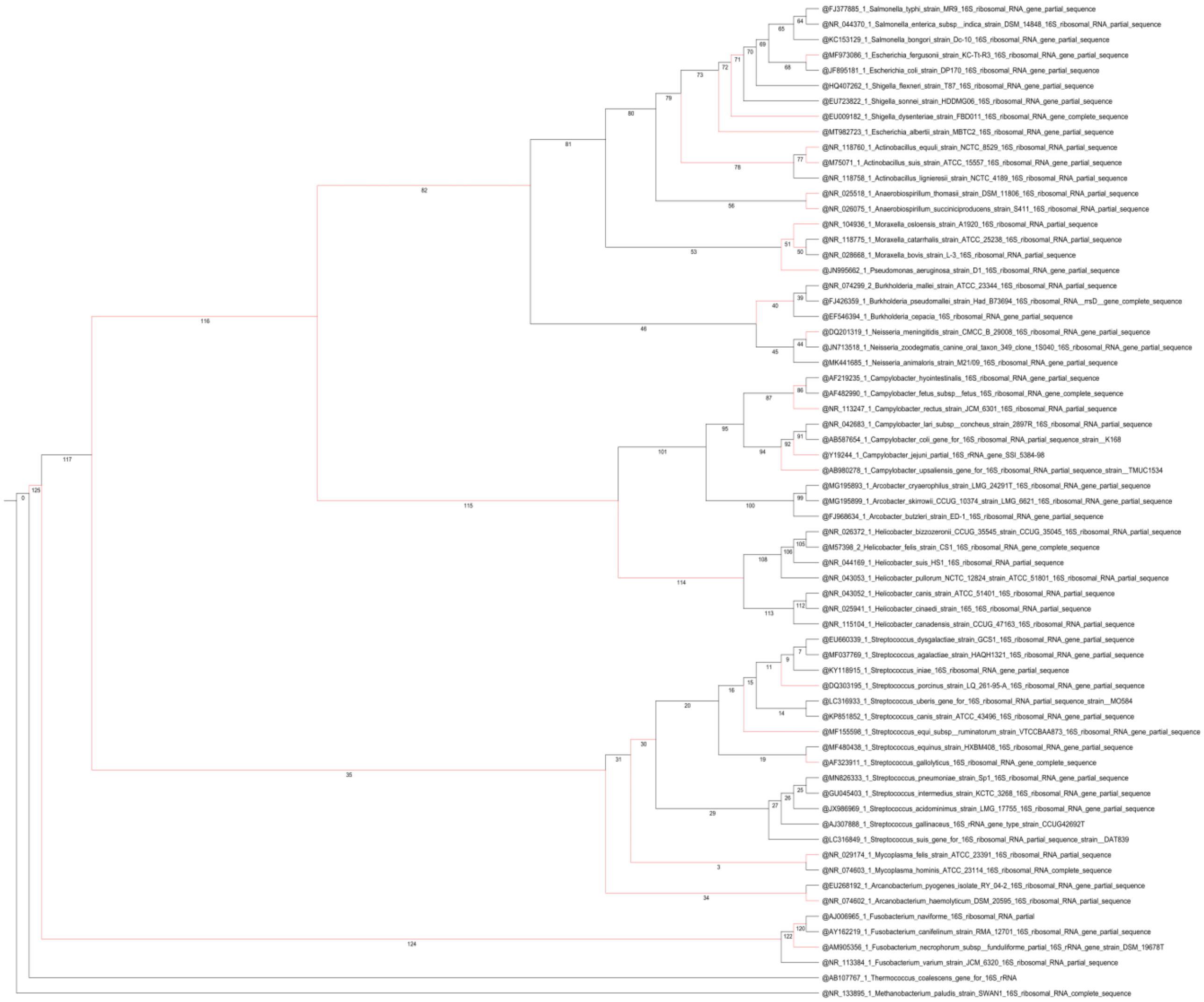

Figure S6: Phylogenetic insertion tree produced by PPLACER using the posterior probability method showing the phylogenetic placements of the sequences obtained with the V8 hypervariable region of the *16S rRNA* gene.

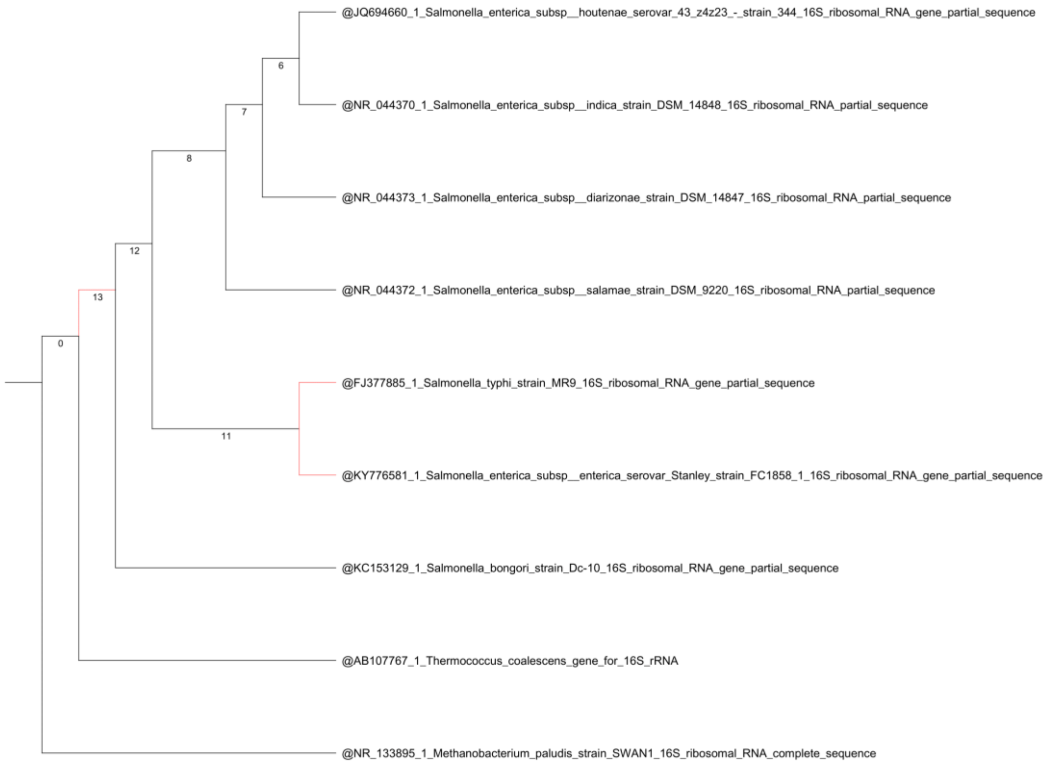

Figure S7: Phylogenetic insertion tree produced by PPLACER using the posterior probability method showing the phylogenetic placements of the sequences obtained with the V9 hypervariable region of the *16S rRNA* gene.
